# A Double Built-In Containment Strategy for Production of Recombinant Proteins in Transgenic Rice

**DOI:** 10.1371/journal.pone.0115459

**Published:** 2014-12-22

**Authors:** Xianwen Zhang, Dongfang Wang, Sinan Zhao, Zhicheng Shen

**Affiliations:** State Key Laboratory of Rice Biology, Institute of Insect Science, College of Agriculture and Biotechnology, Zhejiang University, Hangzhou, China; Deutsches Krebsforschungszentrum, Germany

## Abstract

Using transgenic rice as a bioreactor for mass production of pharmaceutical proteins could potentially reduce the cost of production significantly. However, a major concern over the bioreactor transgenic rice is the risk of its unintended spreading into environment and into food or feed supplies. Here we report a mitigating method to prevent unwanted transgenic rice spreading by a double built-in containment strategy, which sets a selectively termination method and a visual tag technology in the T-DNA for transformation. We created transgenic rice with an inserted T-DNA that harbors a human proinsulin gene fused with the far-red fluorescent protein gene mKate_S158A, an RNAi cassette suppressing the expression of the rice bentazon detoxification enzyme CYP81A6, and an EPSPS gene as the selection marker for transformation. Herbicide spray tests indicated that such transgenic rice plants can be killed selectively by a spray of bentazon at regular field application dosage for rice weed control. Moreover, the transgenic rice seeds were bright red in color due to the fused far-red fluorescent protein, and could be easily visualized under daylight by naked eyes. Thus, the transgenic rice plants reported in this study could be selectively killed by a commonly used herbicide during their growth stage, and their seeds may be detected visually during processing and consumption after harvest. This double built-in containment strategy may greatly enhance the confinement of the transgenic rice.

## Introduction

Transgenic plant as a bioreactor has been presented as a cost effective platform to produce valuable proteins at a large scale [1]. Since the first proof-of-concept report on using plant as a protein expression bioreactor nearly 25 years ago [2], the utility of genetically modified (GM) plants has been expanded to serve as a general platform for the large-scale production of recombinant pharmaceutical proteins and industrial enzymes [3]–[6]. Among the different plants and plant organs, cereal seed has emerged as one of the ideal organs. Cereal seed is the natural organ for protein synthesis and storage, with high protein content, low water content, and low protease activities [7]–[10]. Rice shares the advantages of cereal seed bioreactor such as high grain yield, ease of transformation, and ease of scale-up [11], [12]. Particularly, for the sake of biosafety, rice is a self-pollinating plant and would have a lower risk of unintended gene flow than cross-pollinating plants [13]. A variety of recombinant proteins have been successfully expressed in rice seeds, including human lactoferrin [14], human serum albumin [5], [15], lipase [6], and modified hepatitis B virus surface antigen gene SS1 [16].

As rice (*Oryza sativa* L.) is one of the most important food crops worldwide, the first and foremost concern for the utilization of pharmaceutical transgenic rice is the environmental biosecurity and food safety. Although strict regulation policy and physical containment for bioreactor transgenic rice may greatly reduce the possibility of unintended spreading, accidents could still happen. In fact, several accidents of transgene escapes had been reported in the past several years [17]–[19]. Because transgenic rice and non-transgenic conventional rice are almost identical in appearance, detection of transgenic rice requires sophisticated molecular and biochemical technologies [20]. Thus, if bioreactor transgenic rice mixes with non-transgenic conventional rice unexpectedly, it would be hard to be detected and discovered promptly.

Currently, in addition to physical containment methods, several biological confinement strategies, such as plastid transformation [21]–[23], male sterility [24]–[26], and genetic use restriction technologies (GURTs) [27], [28], were proposed or developed to confine transgene spreading. However, some of these strategies do not move beyond a proof of principle, and some are not suitable for major grain crops such as rice and corn. Thus, an effective and simple strategy that can limit and/or monitor transgenic rice spreading is highly desirable for transgenic rice used for production of recombinant proteins.

We previously reported a built-in strategy to contain transgenic rice [29], [30]. By suppressing the expression of the bentazon detoxification enzyme CYP81A6 [31], we created transgenic rice that can be selectively killed by bentazon, an herbicide commonly used for rice field weed control. This method makes it possible to deselect the transgenic rice plants efficiently by spraying with bentazon.

Red fluorescent protein and Green fluorescent protein (GFP) had been utilized as reporter genes for plants transformation [32]–[35]. Unlike the GFP, which requires UV light for excitation, the far red fluorescent protein mKate_S158A with excitation and emission peaks at 588 nm and 633 nm, respectively, is highly bright under daylight [36]. Thus the far red fluorescent protein expressed in the transgenic plants is visible under daylight by naked eyes.

In this report, we combined the bentazon selective termination strategy with the color tagging method using the far-red fluorescent protein mKate_S158A to create transgenic rice plants that is selectively terminable and visually detectable. We demonstrated in this study that such transgenic rice plants could be selectively terminated by a spray of bentazon and their seeds can be visually detectable by naked eyes under daylight.

## Results

### Creation of transgenic rice bioreactor with safety features

**Figure 1:**
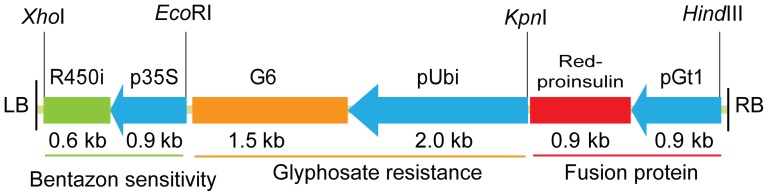
Diagram of the T-DNA for *Agrobacterium* transformation. R450i, the inverted repeat sequence of the 207 bp fragment of *CYP81A6* gene; p35S, cauliflower mosaic virus 35S promoter; G6, the 5-enolpyruvylshikimate-3-phosphate synthase isolated from *Pseudomonas putida* fused with chloroplast transit peptide at the N-terminus (gb: EU169459); pUbi, *Zea mays* polyubiquitin-1 promoter; Red-proinsulin, fusion gene of far-red fluorescent protein mKate_S158A and human proinsulin; pGt1, rice glutelin promoter; LB, left border of the T-DNA; RB, right border of the T-DNA.

To create the transgenic rice bioreactor with a double built-in containment strategy, a binary T-DNA transformation plasmid was constructed based on pCAMBIA1300. Its T-DNA contained a gene encoding a fusion protein of the far red fluorescent protein mKate_S158A and human proinsulin, an *EPSPS* gene for glyphosate tolerance, and an RNAi cassette for expression suppression of the bentazon detoxifying gene *CYP81A6*, respectively (Fig. 1). We transformed this T-DNA into a local rice cultivar “Xiushui134” (*O. sativa japonica*) by *Agrobacterium*-mediated transformation method.

**Figure 2:**
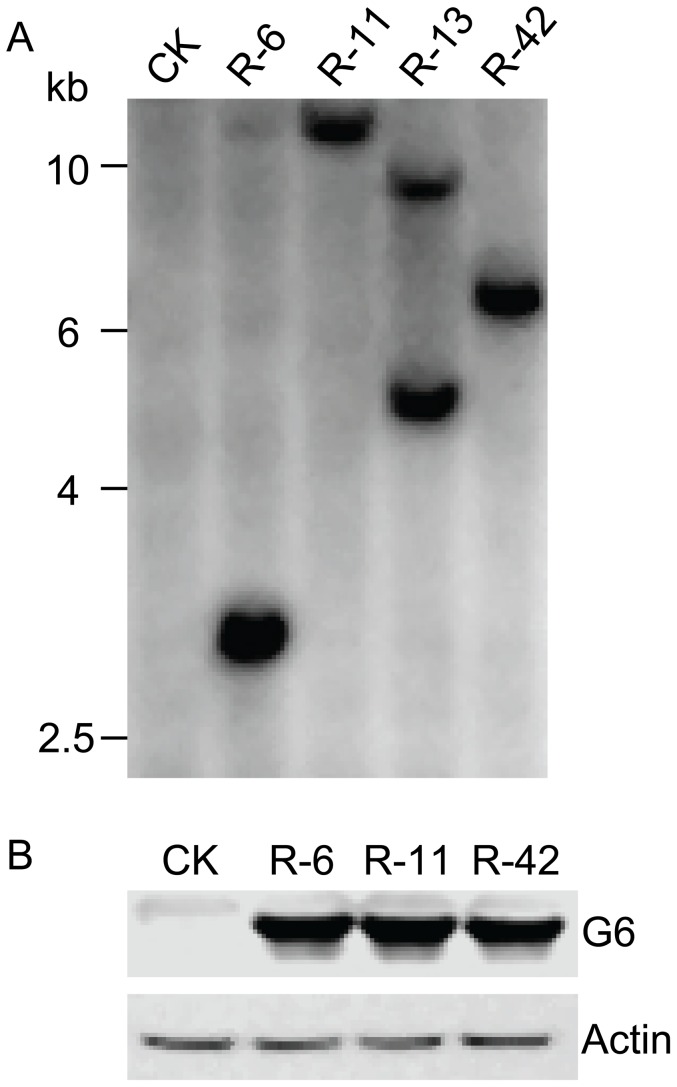
Southern blot and western blot analysis of transgenic rice. A) The genomic DNA was isolated from transgenic rice plants and was hybridized with a probe prepared with DNA encoding mKate_S158A protein. The restriction enzyme used for genomic DNA digestion was *KpnI*. Lanes 1-5, non-transgenic control, event R-6, R-11, R-13 and R-42, respectively. B) Western analysis of G6 (EPSPS) in non-transgenic control (CK) and three independent transgenic events, R-6, R-11 and R-42. Actin was detected by its antibody as loading control.

A total of 235 independent T0 transgenic events were generated using glyphosate as the selection agent. We found that 162 T0 transgenic lines produced visually red seeds. Bentazon spray test showed that about two thirds of these events were sensitive to bentazon. To search for a transgenic event that only had a single copy of the transgene, four events that were sensitive to bentazon with bright red seeds were selected for Southern blot analysis. Among the 4 selected events, the Southern analysis suggested that events R-6, R-11, and R-42 only had a single copy of T-DNA insertion (Fig. 2A). The ration of the number of transgenic and non-transgenic plants among the T1 plants was 3∶1, suggesting that the transgenes in these transgenic lines follow Mendel's law of segregation. Western analysis of the G6 (EPSPS) protein in all the transgenic rice plants of these 3 events showed a specific band of about 45 kDa, which is the expected size of the G6 (EPSPS) protein (Fig. 2B). This result is consistent with the observation of their tolerance to glyphosate. Homozygous plants of these three events were identified and selected from T1 plant population for further characterization.

### Visual detection of transgenic rice seeds

**Figure 3:**
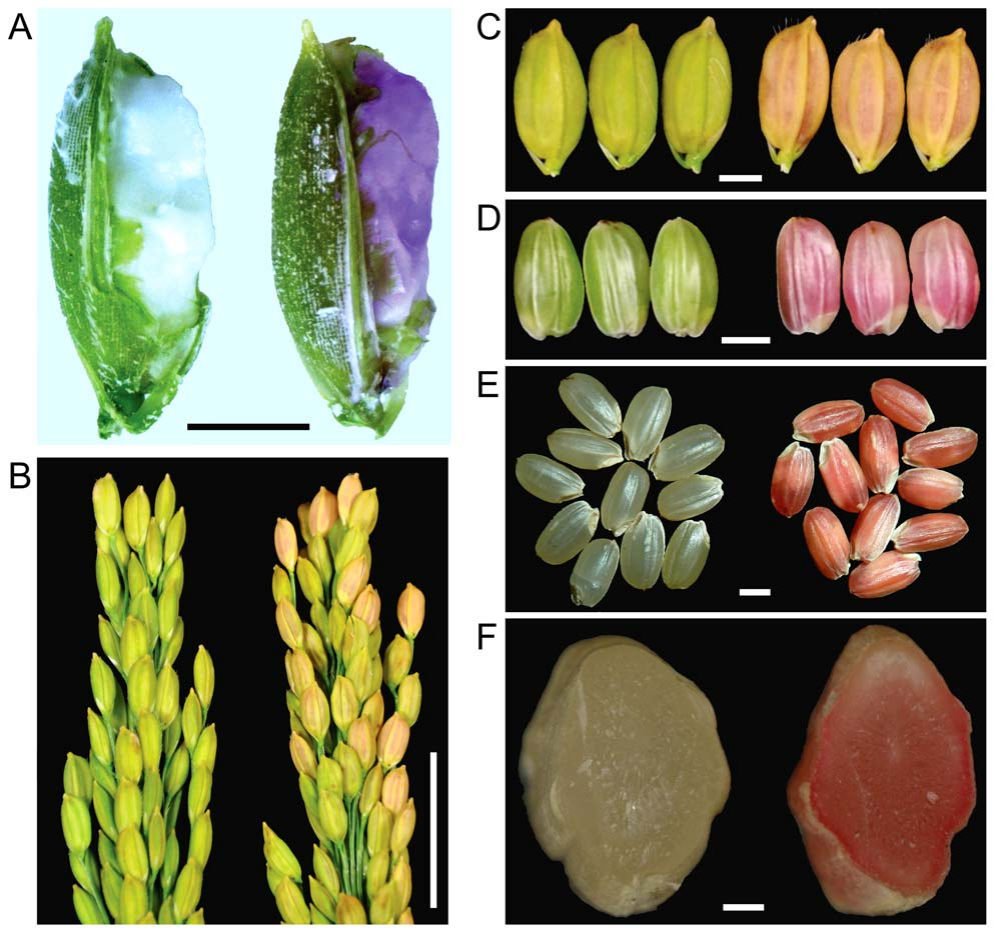
Visual detection of the far-red fluorescent fusion protein of the transgenic rice seeds. XS-134 is a *japonica* rice cultivar and served as the recipient of transformation; R-42 is a transgenic rice event. The samples in (A-G) were in the same order (Left, XS134; Right, R-42). A) Rice seeds in milk-ripe stage. Scale bar, 2 mm; B) Panicles of rice in wax ripe stage. Scale bar, 2 cm; C) Rice seeds in wax ripe stage. Scale bar, 2 mm; D) Rice seeds after dehusking in wax ripe stage. Scale bar, 2 mm; E) Ripened rice seeds. Scale bar, 2 mm; F) Cross section of ripened rice seeds. Scale bar, 500 µm.

To monitor the far-red fluorescence in transgenic rice seeds, we visually observed the spikelet of T2 transgenic rice plants daily after pollination. When the transgenic rice seeds grew from filling stage to milk-ripe stage, the transgenic rice seeds began to present purple reddish color, which was quite different visually from the non-transgenic rice seeds (Fig. 3A). When the seeds grew into waxy ripe stage, the red color could be observed clearly with or without dehusking (Fig. 3B, 3C, 3D). After the rice seeds grew to complete ripeness, the red color of the hulled seeds was even brighter (Fig. 3E). This bright red was observed in the whole part of the endosperm (Fig. 3F). Clearly, the red color of the transgenic seeds is distinct enough to be monitored from milk-ripe stage to complete ripeness by naked eyes. We checked the transgenic rice seeds with 1 year of storage time at room temperature, and found the red color was still as bright.

### Selective termination of the transgenic rice plants

**Figure 4:**
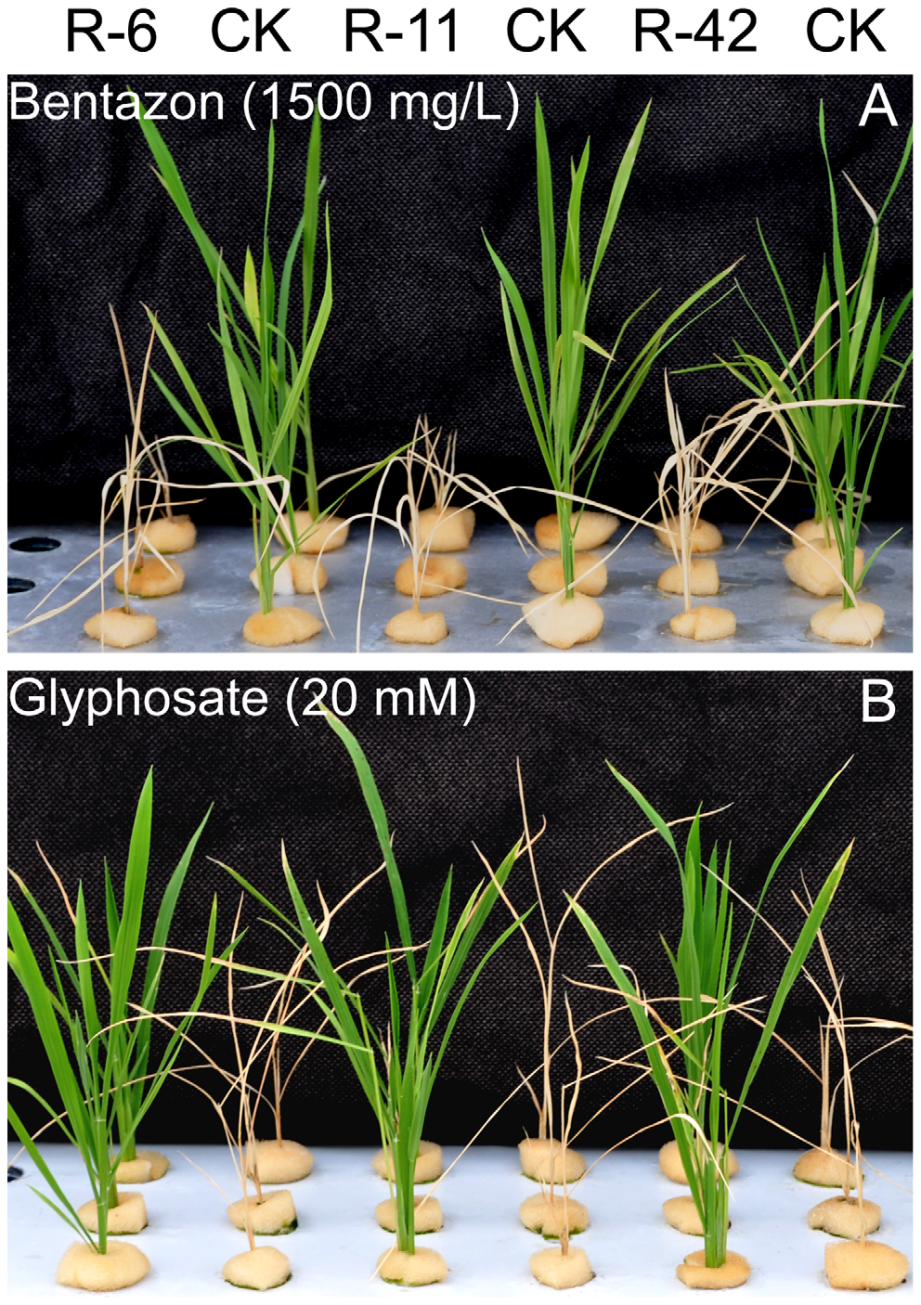
Sensitivity test of transgenic rice plants to bentazon and glyphosate. The T2 transgenic rice events (R-6, R-11 and R-42) along with non-transgenic ones (CK) were cultured in greenhouse and sprayed with 1500 mg/L bentazon (A), or 20 mM glyphosate (B). The plants in panel A and B were in the same order.

The T2 homozygous plants of the 3 independent transgenic events R-6, R-11, R-42 were cultured in hydroponics in the greenhouse and analyzed for their sensitivity to bentazon and glyphosate. One group of plants was sprayed with bentazon at 1500 mg/L, and the other group of plants with glyphosate at 20 mM. After 10 days, we found that the bentazon spray killed all the transgenic rice plants but not the non-transgenic plants (Fig. 4A), while the glyphosate spray killed all non-transgenic plants but not the transgenic plants (Fig. 4B). This test demonstrated that the transgenic rice plants could be selectively terminated by bentazon.

**Figure 5:**
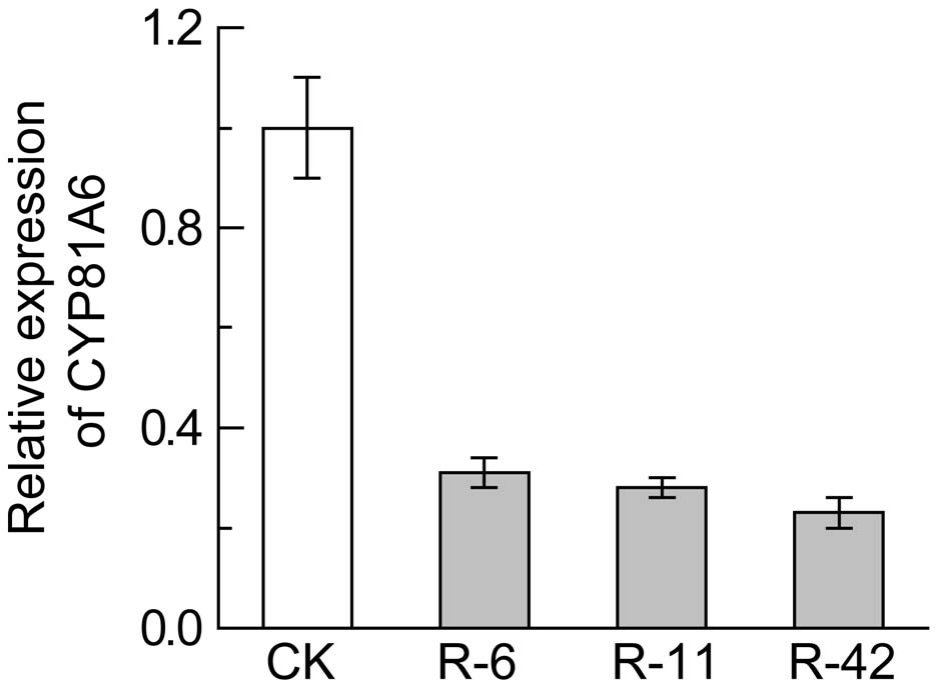
Analysis of the expression level of the *CYP81A6* gene. Relative expression levels of *CYP81A6* were measured by real-time PCR in non-transgenic control (CK) and three independent transgenic events R-6, R-11 and R-42, respectively. Values are means ± SD of three independent experiments.

To investigate the relationship between bentazon sensitivity and the suppression of the *CYP81A6* mRNA in transgenic rice plants, the mRNA of *CYP81A6* in the plants of the three independent transgenic events R-6, R-11 and R-42 were measured by quantitative RT-PCR (qRT-PCR). The results indicated that the transcript levels of *CYP81A6* in the transgenic plants of all the three events were significantly lower than in the non-transgenic control plants (Fig. 5), suggesting that the sensitivity to bentazon were likely caused by the suppression of the *CYP81A6* expression in the transgenic plants.

### Analysis of the fusion protein in transgenic rice seeds

**Figure 6:**
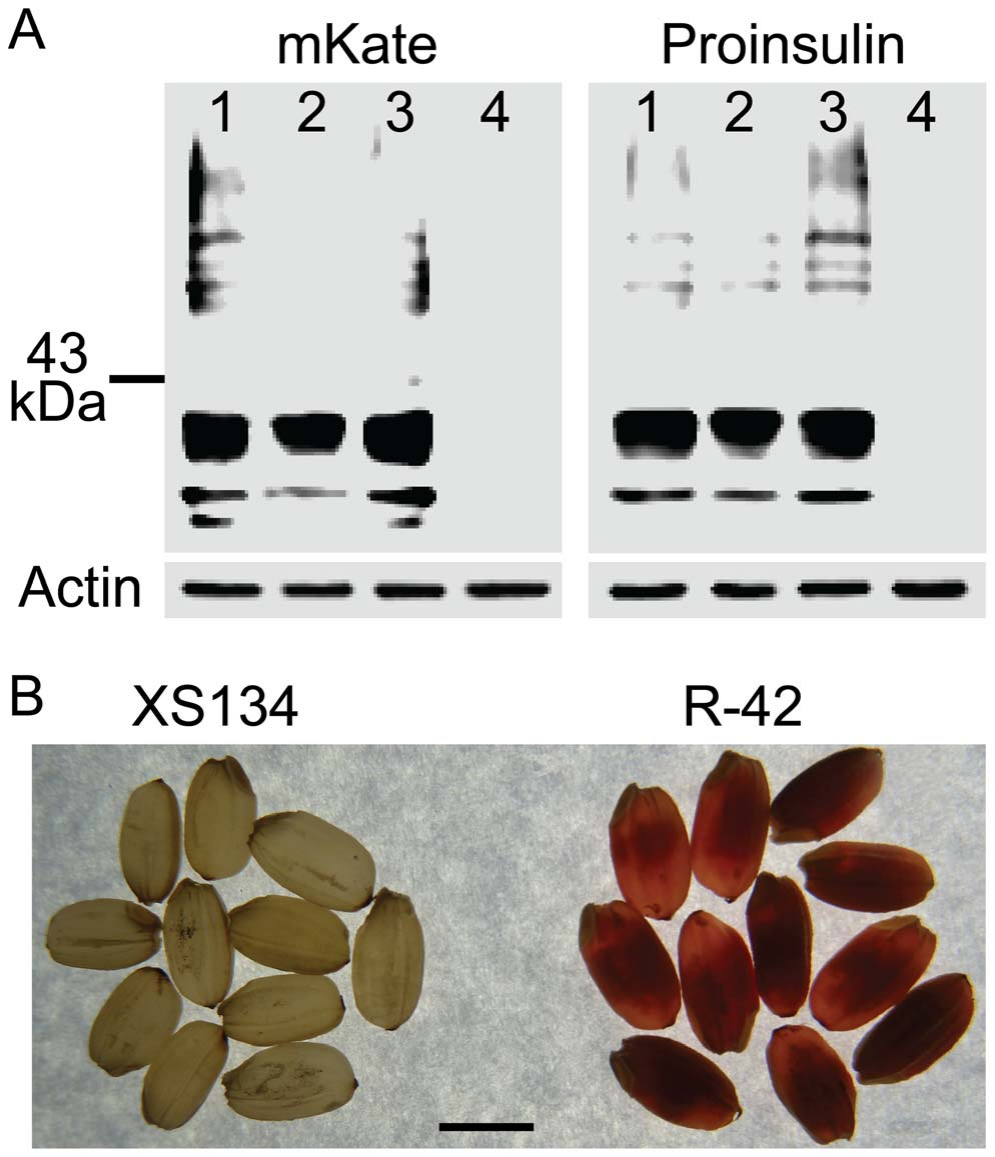
Analysis of the fusion protein in transgenic rice seeds. A) Protein levels of fusion protein of mKate and human proinsulin in non-transgenic control and three independent transgenic events R-6, R-11 and R-42. Left panel, protein blot with the antibody to mKate; Right panel, protein blot with the antibody to proinsulin. In both left and right panels, using protein blot with the antibody to plant actin as loading control. Lanes 1-4, event R-6, R-11, R-42 and non-transgenic control, respectively. B) Transparency of ripened rice seeds. XS-134, non-transgenic control; R-42, a transgenic rice event. Scale bar, 2 mm.

In order to examine the expression of the proinsulin fusion protein, we carried out western blot analysis for the seed extracts of the transgenic rice with a monoclonal antibody specific to human proinsulin or mKate. A strong signal with an estimated molecular mass closed to the calculated size of the fusion protein (36 kDa) was detected in all of the transgenic events but not in the non-transgenic control plants with antibody against either human proinsulin or mKate (Fig. 6A). Weak bands of bigger or smaller size were also observed, and they were likely resulted from cross-link or degradation of the fusion protein. This result suggested that the human proinsulin fusion protein was highly expressed with the expected size in the transgenic rice seeds. As the far-red fluorescent protein was fused to the proinsulin, the brightness of the red color in the endosperm should be a direct indicator of the expression level.

### Comparison of agronomic traits

We compared the major agronomic traits among the transgenic rice plants and the non-transgenic ones, and found no significantly difference in plant height, panicles per plant and number of grains per panicle [Table 1]. However, the transgenic rice seeds had lower 1000-grain weight [Table 1], and were also less transparent than the non-transgenic control seeds (Fig. 6B). These phenotypes in seed were also observed in the seeds expressing a cellulase or a lipase [6], [37]. The alteration of the seed transparence is likely due to the over-expression of exogenous proteins in endosperms.

**Table 1:** Comparison of agronomic traits of the transgenic and non-transgenic rice plants (CK) under field conditions.*

Traits	CK	R-6	R-11	R-42	ANOVA		
					*F*	*P*	df
Plant height (cm)	71.4±2.0^a^	70.9±2.1^a^	70.6±2.3^a^	70.5±2.2^a^	1.121	0.344	119
Panicles per plant	15.4±3.1^a^	15.1±2.9^a^	14.9±2.9^a^	14.8±2.4^a^	0.213	0.888	119
Grains per panicle	122.2±5.1^a^	123.7±6.0^a^	119.8±5.4^a^	120.8±5.5^a^	2.891	0.038	119
1000-grain weight (g)	25.8±0.4^a^	25.6±0.2^a^	24.5±0.2^b^	23.2±0.3^c^	74.720	0.000	15

^*^ Letters (a, b or c) following the values (means ± SD) in the same row indicate significant differences among different rice plants. Values with the same letter are not significantly different from each other (LSD test, P<0.05).

## Discussion

The risk of transgene spreading is now a major obstacle for commercial production of recombinant protein using transgenic rice. Transgene spreading may occur at cultivation phase via pollen [38]–[40], or in the process of harvest, transport, processing, purification, packaging, storage and disposal [41], [42]. As rice is planted by large number of small farmers worldwide as a staple food for more than half of the world population, a rice bioreactor transgene will be hard to be detected, let alone to be eliminated if it is escaped to these small farms. Therefore, regular control methods such as physical isolation may not be sufficient to ensure the total control of bioreactor rice. Novel methods to mitigate the risk of transgene spreading are highly desirable.

**Figure 7:**
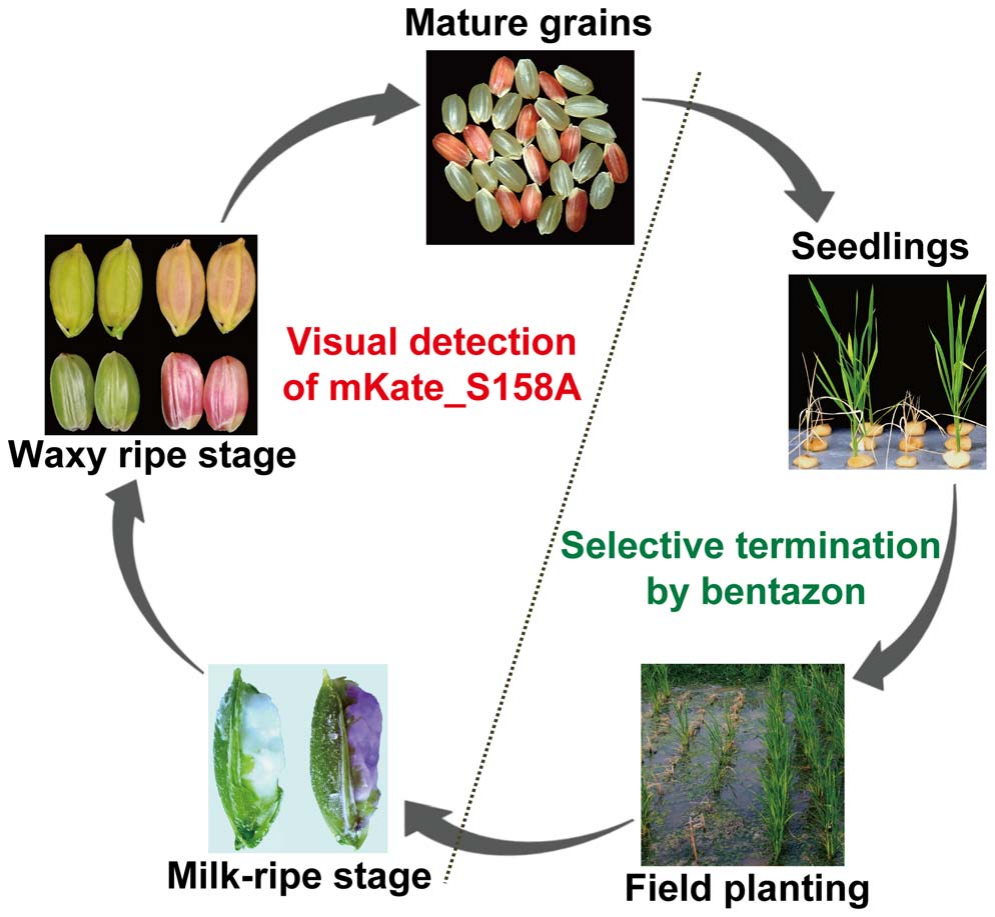
Illustration of the double built-in containment strategy for pharmaceutical transgenic rice. Bentazon is used for selective termination of transgenic rice during field planting stage as reported previously [29]. Visual detection can be used to monitor the transgenic rice seeds after harvest.

The double built-in containment strategy reported in this study may greatly reduce the risk of transgene spreading. This strategy provides control measures for planting phase as well as seed processing and consumption phase (Fig. 7). At planting stage, the unintended transgenic bioreactor rice plants can be effectively terminated preemptively by spray of bentazon. As bentazon is commonly used for rice weed control, application of bentazon in regular rice will ensure the elimination of transgenic bioreactor rice without incurring significant extra cost. Especially, the rice field with proximity to the rice bioreactor farm may be recommended to use bentazon for weed control. During rice processing and consumption phase, any contaminated transgenic bioreactor seeds would be easily detected by their red color. Importantly, this visual detection feature could enable rice consumers to be their own contamination examiners, which could build a huge confidence for rice consumers on the safety of their rice. However, the double built-in technology reported here was not intend to substitute or eliminate the regular physical spreading control. It is intended to provide extra layer of safety to ensure the safe use of the transgenic rice for recombinant protein production.

In this study, the far red fluorescent protein mKate_S158A was fused to human proinsulin to visualize the recombinant protein. This method may be preferred if the recovery of the target protein from the fusion protein is technically feasible and cost-effective. Alternatively, an independent expression cassette of the far red fluorescent protein may also be linked tandemly to the expression cassette of the protein of interest. This two cassette method will produce individual protein of interest directly, which will likely result to cost reduction for protein purification and processing. However, the expression levels of two tandemly linked genes are often different, and it will be likely much harder to obtain a transgenic event with both genes highly expressed. Moreover, the chance of separation of the two cassettes by recombination, although at very low level, is much higher than the chance of division of the fusion gene functionally [43], [44].

New technologies may be utilized to take the advantages of both fusion strategy and two cassette strategy. One is the protein splicing technology, in which a fusion protein was produced and automatically spliced into two proteins during purification [45], [46]. The other technology is to use a polycistronic transgene with a self-cleavage peptide 2A [47]–[50].

The proinsulin was used as an example in this study to illustrate the utilization of this novel double built-in confinement technology. This technology could certainly be also used for production of other high value proteins, such as pharmaceutical proteins and industrial enzymes. Although the proinsulin fusion protein appeared to be highly expressed in the transgenic seeds we obtained and studied, the feasibility for mass production of insulin from these seeds still needs to be assessed by further studies on purification and processing of the fusion protein.

## Materials and Methods

### Construction of binary vector for rice transformation

The binary plasmid named p1300-450i-G6 was described previously [13], which contained a 207 bp inverted repeat sequence of *CYP81A6* for RNA interference and an *EPSPS* gene *G6* conferring glyphosate tolerance (gb: EU169459). Briefly, the 207 bp fragment of the 5′ end of *CYP81A6* was obtained by PCR from rice genomic DNA using the primer 450F (5′CTCGAGCAGTGCACCAGAGTCACAGAAACACATCACAC, an *Xho*I site was attached and underlined), and 450R (5′ AGATCT GCTTCTTGACGAGGTGGAGGTGT, a *Bgl*II site was attached and underlined). This fragment represents the 5′ end of *CYP81A6* cDNA from 1 to 207 bp. Another 327 bp fragment of the 5′ end of *CYP81A6* was obtained by PCR from the same rice genomic DNA using the primer 450F, and the primer 450R2 (5′ AGATCT CGGTGAAGCACTCCCTGGCGCAC, a *Bgl*II site was attached and underlined). This fragment represents the very 5′ end of the cDNA from 1 to 327 bp. Both PCR products were cloned into the pMD-T vector (Shanghai Sangon, China), and then released from the T-vectors by digestion with *Xho*I and *Bgl*II. These two fragments were cloned into the T-DNA plasmid vector pCAMBIA1300 which was predigested with *Xho*I and dephosphorylated. The resulted plasmid, which contains a 207 bp inverted repeat sequence of *CYP81A6* for RNA interference, was named as p1300-450i.

The *Zea mays* polyubiquitin-1 promoter (ZmUbi-1) was obtained by PCR using ZmUbiF-K (5′ TGGGTACCGTGCATGCCTACAGTGCAGCGTGACCCGGTCGTGC, a *Kpn*I site was attached and underlined) and ZmUbiR (5′ GTGGGATCCTCTAGAGTCGACCTGCAGAAGTAACAC CAAACAACAG, a *BamH*I site was attached and underlined). ZmUbi-1 was used for the direct expression of the *G6* gene fused with the chloroplast transit peptide from the acetohydroxyacid synthase of *Z. mays*. The PCR amplified ZmUbi-1 promoter was digested with *BamH*I and *Kpn*I, and then ligated in a 3-way to the synthetic *G6* gene predigested with *EcoR*I and *BamH*I, and the plasmid p1300-450i predigested with *EcoR*I and *Kpn*I. This binary plasmid was named as p1300-450i-G6.

A 884 bp sequence which encode the fusion protein of the far-red fluorescent protein mKate_S158A (gb: EU383029) and human proinsulin fragment (gb: AGC54790) was synthesized by Shanghai Sangon Limited Corp (Shanghai, China), with the following modifications: an *Xba*I site was introduced at the 5′ end of the fusion gene; a corn phosphoenolpyruvate carboxylase (PEPC) terminator with a *Kpn*I site was added to the 3′ end of the fusion gene. The promoter and the signal peptide of rice storage protein Gt1 [6] was obtained by PCR using two primers, Gt1F (5′ AAGCTT TTGGAAAGGTGCCGTGC AGTT, a *Hind*III site was attached and underlined) and Gt1R (5′ TCTAGA CTGGGCTAGGGAGCCAT CGCACAAG, an *Xba*I site was attached and underlined). The PCR product was first cloned into pMD18-T vector and sequenced. A three-way ligation was carried out with p1300-450i-G6 predigested with *Hind*III and *Kpn*I as the vector backbone. One insert was Gt1 promoter (including the signal peptide) predigested with *Hind*III and *Xba*I, and the other insert was the far-red fluorescent protein fused human proinsulin fragment predigested with *Xba*I and *Kpn*I. The final binary T-DNA construct was named as p1300-450i-G6-red-proinsulin and was used for *Agrobacterium* mediated rice transformation.

### Agrobacterium-mediated rice transformation

T-DNA transformation plasmid vector p1300-450i-G6-red-proinsulin was transformed into *Agrobacterium tumefaciens* (LBA4404) by electroporation. A local rice cultivar “Xiushui-134” (*O. sativa japonica*) was transformed using an *Agrobacterium*-mediated transformation method described previously [51]. Glyphosate (Sigma) of 2∼3 mM final concentration was used for selection and regeneration of transgenic calli. The independently transformed events were cultured in the greenhouse in solution prepared according to Yoshida et al. (1976) at about 18–25°C with 12–14 h light [52].

### Selection of homozygous plant

T1 plants of the transgenic event R-6, R-11 and R-42 were planted individually. The homozygous plants were identified by checking if any segregation of the transgene in its T2 plant population. The homozygous plants were used for the characterization in this study.

### Spray of herbicides

The target rice plants in tillering stage were sprayed with handhold sprayer at the rate of 100 mL/m^2^ for bentazon and glyphosate. Bentazon (48% solution) was obtained from Jiangsu Luli Limited (Jiangsu, China) and sprayed with the final concentration of 1500 mg/L. To test glyphosate tolerance, Roundup (41% propylamine salt of glyphosate, Monsanto, USA) was used. It was diluted to 20 mM and then added with Tween-20 to the final concentration of 0.01% for spray, and the growth of the plants was monitored daily.

### RNA extraction, cDNA isolation and quantitative RT-PCR (qRT-PCR)

Three transgenic rice plants from different transgenic events and non-transgenic control plants of the same cultivar were sampled at 30 days after germination. Total RNA was extracted from 100 mg of leaves using SV Total RNA Isolation System kit (Promega). The same amount of RNA was converted into single-strand cDNA using a PrimeScript RT reagent kit (TaKaRa, Japan). For comparison of the transcript of CYP81A6 between the transgenic plants and the non-transgenic control, PCR amplification products generated by using the same single-strand cDNA as the template were analyzed by 1% agarose gel electrophoresis. Rice Ubiquitin gene was used as an internal control. The primers used for qRT-PCR were R450-qF (5′ GGCGAGAAGAAGAGCATGAT) and R450-qR (5′ GACATCGCCCATTCTGATGT). qRT-PCR was performed using a SYBR Green RT-PCR kit (BIO-RAD) with an ABI PRISM 7500 sequence detection system (Applied Biosystems). For each qRT-PCR experiment, three technical repetitions were performed, and the mean values were calculated.

### Western blot analysis

Standard western blot analysis method was carried out. For detection of the expression of fusion protein of mKate_S158A and human proinsulin in transgenic rice seeds. Transgenic rice seeds as well as non-transgenic control rice seeds were ground to powder and then suspended in SDS loading buffer. After lysis and centrifugation, the soluble fractions of these samples were separated by 10% SDS-PAGE and then blotted onto nitrocellulose membrane. The mouse antiserum against human proinsulin (Abcam, Cat. No. ab76570) or mKate (ORIGENE, Cat. No. TA180091) was used as the first antibody and the alkaline phosphatase-conjugated goat anti-mouse IgG (Sigma, Cat. No. A3562) as the second antibody. For detection of the expression of G6 (EPSPS) protein of plant leave, the rabbit antiserum against G6 was used as the first antibody and the alkaline phosphatase-conjugated goat anti-rabbit IgG as the second antibody (Sigma, Cat. No. A3687). For getting the the rabbit antiserum against G6, the cDNA of G6 gene was cloned into pET28b (Novagen) for *E. coil* expression. The recombinant G6 protein was gel purified and used to immunize rabbits. The resulting G6 antibodies were purified through an IgG affinity chromatography column prior to use. Plant actin protein as loading control. For detection of the expression of plant actin protein, the rabbit antiserum against plant actin (EARTHOX, Cat. No. E021080) was used as the first antibody and the alkaline phosphatase-conjugated goat anti-rabbit IgG (Sigma) as the second antibody (Sigma). Protein blots were visualized using the SuperSignal Ultra ECL chemiluminescence kit (MultiSciences Biotech, Hangzhou, China) according to the manufacturer's protocol.

### Southern blot analysis

Southern blot was carried out according to the DIG System Manual (Roche, Cat. No. 11585614901). Genomic DNA was isolated from rice leaves using the CTAB-based method [53]. 50 µg genomic DNA was digested with *Kpn*I and size-fractionated on a 0.7% (w/v) agarose gel by electrophoresis. The denatured and neutralized DNA was then transferred to nylon membranes (Hybond-N^+^, Amersham, UK) using the capillary transfer method. The hybridization probe specific to *mKate_S158A* gene was prepared according to the DIG System Manual (Roche). The mKate_S158A probe was amplified by PCR using primers Red-F (5′ GCATTTAGGTTGTGATGAAGTCCGAACTCATCACCGAGA) and Red-R (5′CTCGAGCTCTCATTACGGCTTCTCGCCCTCCTCGCGCTTG). The blot was hybridized with the probe at 55°C for overnight, and then washed with 2×SSC, 0.1% SDS at 25°C for 10 min. A second wash with 0.5×SSC, 0.1% SDS at 65°C for 30 min was followed.

### Field trials

Both the transgenic and non-transgenic control rice plants were planted and tested in field at the Zhejiang University Farm in Hangzhou, China. At harvest time, the agronomic traits on plant height, number of panicles per plant, grains per panicle and 1,000-grain weight were measured, recorded and the mean values were calculated.
